# Ubiquitin–Proteasome System Is Required for Efficient Replication of Singapore Grouper Iridovirus

**DOI:** 10.3389/fmicb.2018.02798

**Published:** 2018-11-26

**Authors:** Xiaohong Huang, Shina Wei, Songwei Ni, Youhua Huang, Qiwei Qin

**Affiliations:** ^1^College of Marine Sciences, South China Agricultural University, Guangzhou, China; ^2^Key Laboratory of Tropical Marine Bio-Resources and Ecology, South China Sea Institute of Oceanology, Chinese Academy of Sciences, Guangzhou, China; ^3^Laboratory for Marine Biology and Biotechnology, Qingdao National Laboratory for Marine Science and Technology, Qingdao, China

**Keywords:** iridovirus, ubiquitin, proteasome, viral replication, grouper

## Abstract

The ubiquitin–proteasome system (UPS) serves as the major intracellular pathway for protein degradation and plays crucial roles in several cellular processes. However, little is known about the potential actions of the UPS during fish virus infection. In this study, we elucidated the possible roles of UPS in the life cycle of Singapore grouper iridovirus (SGIV); a large DNA virus that usually causes serious systemic diseases with high mortality in groupers. Data from transcriptomic analysis of differentially expressed genes illustrated that expression of 65 genes within the UPS pathway, including ubiquitin encoding, ubiquitination, deubiquitination, and proteasome, were up- or down-regulated during SGIV infection. Using different proteasome inhibitors, inhibition of the proteasome decreased SGIV replication *in vitro*, accompanied by inhibition of virus assembly site formation, and viral gene transcription and protein transportation. Over-expression of ubiquitin partly rescued the inhibitory effect of ubiquitin inhibitor on SGIV replication, suggesting that UPS was required for fish iridovirus infection *in vitro*. Viral or host proteins regulated by proteasome inhibition during SGIV infection were investigated with two-dimensional gel electrophoresis and matrix-assisted laser desorption/ionization time-of-flight mass spectrometry. Sixty-two differentially expressed proteins, including 15 viral and 47 host proteins, were identified after SGIV infection. The host proteins were involved in ubiquitin-mediated protein degradation, metabolism, cytoskeleton, macromolecular biosynthesis, and signal transduction. Among them, 11 proteins were negatively regulated upon MG132 treatment during SGIV infection. This is believed to be the first study to provide evidence that UPS was essential for fish virus infection and replication.

## Introduction

The ubiquitin-proteasome system (UPS) is the major intracellular protein degradation pathway and plays crucial roles in a variety of fundamental cellular processes, including regulation of gene transcription, cell cycle progression, autophagy, development and differentiation, and modulation of the immune and inflammatory responses (Glickman and Ciechanover, 2002; Wertz and Dixit, 2008; Zhao and Goldberg, 2016). There is increasing evidence that the UPS is required for viral infection by affecting viral entry, gene transcription, assembly, release and immune evasion (Banks et al., 2003; Wang et al., 2016; Casorla-Pérez et al., 2017). To the best of our knowledge, DNA viruses, as well as RNA viruses from different hosts, including mammals, insects, and plants, exploit the UPS system at various stages of the viral life cycle (Camborde et al., 2010; Contin et al., 2011; Katsuma et al., 2011; Greene et al., 2012; Wang et al., 2016). The proteasome machinery seems to play opposing roles during viral infection. On the one hand, proteasome inhibition with bortezomib leads to increased susceptibility to lymphocytic choriomeningitis virus or coronavirus infection *in vivo* (Basler et al., 2009; Raaben et al., 2010). On the other hand, inhibition of proteasome activity prevents viral DNA replication and the formation of virus assembly sites during vaccinia virus (VACV) replication (Satheshkumar et al., 2009). Inhibition of proteasome activity also reduces Kaposi’s sarcoma-associated herpesvirus (KSHV) entry into endothelial cells and intracellular trafficking (Greene et al., 2012). Therefore, exploration of the molecular mechanism by which the UPS regulates viral replication will provide an alternative potential target for antiviral therapy.

Singapore grouper iridovirus (SGIV), a novel member of the genus *Ranavirus*, family *Iridoviridae*, was first isolated from diseased groupers. SGIV infection causes >90% mortality in groupers and sea bass (Qin et al., 2001). Our previous studies demonstrated that SGIV infection in grouper cells induces non-apoptotic cell death, and mitogen-activated protein kinase (MAPK) signaling pathways, including extracellular signal-regulated kinase, p38 MAPK, and c-Jun N-terminal kinase signaling, which are involved in viral replication (Huang et al., 2011a,b). Genome annotation of SGIV reveals that some potential viral gene products, including ubiquitin (ORF102L) and predicted E3 ubiquitin ligase (ORF146L), might be involved in the regulation of the UPS during SGIV infection (Song et al., 2004). Transcriptome analysis of SGIV-infected grouper spleen shows that several genes associated with ubiquitin-mediated proteolysis are up- or down-regulated in response to SGIV infection, suggesting that the UPS plays important roles in SGIV infection (Huang Y.H. et al., 2011). However, the molecular mechanism underlying the regulatory effects of UPS on SGIV replication remain uncertain.

In this study, we explored the importance of the UPS in SGIV infection using different proteasome inhibitors. Moreover, viral or cellular proteins regulated by the UPS pathway during SGIV infection were investigated with two-dimensional gel electrophoresis (2-DE) and matrix-assisted laser desorption/ionization time-of-flight mass spectrometry (MALDI-TOF MS). This study is believed to be the first to show molecular evidence that the UPS is involved in fish iridovirus infection, providing new clues to understanding the fish-virus interaction.

## Materials and Methods

### Materials

Proteasome inhibitors MG132 (carbobenzoxy-L-leucyl-L-leucyl-L-leucinal) and lactacystin were purchased from Sigma. Bortezomib and ubiquitin-activating enzyme (E1) inhibitor (Pyr-41) were purchased from Selleckchem. These inhibitors were dissolved in dimethyl sulfoxide (DMSO), and their cytotoxicity on grouper spleen (GS) cells was determined using trypan blue exclusion dye staining.

### Cells and Virus

The GS cell line used in this study was established in our laboratory (Huang et al., 2009). GS cells were cultured in Leibovitz’s L-15 supplemented with 10% fetal bovine serum (Gibco) and kept in incubators at 25°C. SGIV was propagated in GS cells and stored at -80°C. For the inhibition experiments, GS cells were pretreated with DMSO or various concentrations of inhibitors for 2 h, and then infected with SGIV at multiplicity of infection (MOI) of two for indicated times.

To explore which steps of SGIV replication are affected by proteasome disruption, virus production was determined after treatment with MG132 at different time points during SGIV infection as described previously (Chen et al., 2008). MG132 treatment was carried out at different time points during infection, and then cells were washed to remove MG132 for further incubation until 24 h. In detail, GS cells were pre-treated with MG132 for 2 h, and replaced with normal medium in “P” group. In “TH” or “DMSO” group, MG132 or DMSO was present in the medium throughout virus infection. In addition, MG132 was present in the medium only for 0–6 h, 6–12 h, 12–18 h, and 18–24 h in “0–6 h,” “6–12 h,” “12–18 h,” and “18–24 h” groups, respectively. Finally, the whole cell lysate at indicated time points were collected and determined for virus titration as described below.

### Viral Titer Assay

Viral titers were determined on monolayers of GS cells in 96-well plates with 50% tissue culture infective dose (TCID_50_) assay as described previously (Huang et al., 2011a). SGIV was serially diluted 10-fold and overlaid on ∼95% confluent monolayers of GS cells in 96-well plates and incubated for 1 h. After removing the medium containing virus, cells were washed with fresh medium three times. Finally, cells were incubated with fresh medium and cultured at 25°C for 5 days. The cytopathic effect (CPE) was observed under microscopy and virus titer (TCID_50_/ml) was calculated according to Reed and Muench (1938). The results were expressed as means of three independent experiments. The statistical significances were determined with Student’s *t*-test. The significance level was defined as *p* < 0.05 (^∗^).

### RNA Sequencing and Analysis

To explores the expression profiles of host genes in response to SGIV infection, RNA sequencing was carried out in SGIV infected GS cells. In brief, mock- or SGIV-infected GS cells (12, 24, and 48 h p.i.) in triplicate flasks were collected and total RNA was extracted using the mirVana miRNA Isolation Kit (Ambion) following the manufacturer’s protocol. The libraries were constructed using TruSeq Stranded mRNA LT Sample Prep Kit (Illumina, San Diego, CA, United States) and then sequenced on the Illumina sequencing platform (HiSeqTM 2500). After the initial assembly, the differentially expressed genes (DEGs) related to UPS was analyzed as described previously (Gao and Chen, 2018). The changes of target genes were analyzed using the expression levels in SGIV-infected cells compared to those in mock-infected cells at indicated time points.

### Electron Microscopy

DMSO- or MG132-treated GS cells were infected with SGIV and harvested at 24 h post-infection (h p.i.). Sample preparation was performed as previously described (Huang et al., 2011a). After washing with phosphate-buffered saline (PBS), cells were post-fixed in 1% osmium tetroxide for 1 h, and then dehydrated in graded ethanol. The cells were embedded in EPON resin. Sections were double stained with uranyl acetate and lead citrate. The ultrathin sections were examined in a JEM-1400 electron microscopy (Jeol) at 120 kV.

### Immunofluorescence Assays

GS cells were grown on coverslips in six-well plates, and then infected with SGIV at MOI 2 in the presence or absence of 10 μM MG132. At 24 h p.i., the infected cells were fixed in 4% paraformaldehyde and permeabilized with absolute alcohol for 7 min at -20°C. After washing with PBS, the cells were blocked with 2% bovine serum albumin for 30 min, and then incubated with primary antibodies (anti-VP19 serum 1:100) for 2 h at room temperature. The coverslips were washed with PBS, followed by incubation with the secondary antibody, fluorescein-isothiocyanate-conjugated goat anti-mouse IgG (1:100, Pierce) for 1 h. Nuclei were stained with 1 μg/ml 4′,6-diamidino-2-phenylindole (DAPI). Samples were observed under an inverted fluorescence microscope (Leica).

### Quantitative Real-Time PCR Analysis (qRT-PCR)

To confirm the effects of MG132 on viral gene expression, qRT-PCR was used to evaluate the relative RNA expression of several genes. The specific PCR primers for the viral genes are described previously (Huang et al., 2011b). Total cell RNA was extracted from DMSO- or MG132-treated infected cells at 6, 12, and 24 h p.i. RNA extraction was performed using SV Total RNA Isolation Kit (Promega). RNA reverse transcription was carried out using ReverTra Ace qPCR RT Kit (Toyobo) according to the manufacture’s instruction. qRT-PCR was performed in a Roche 480 Real Time Detection System (Roche, Germany) using the SYBR Green Real-time PCR Kit (Toyobo) as described previously (Huang et al., 2011b). Each assay was performed in triplicate, and β-actin was chosen as the internal control. The data are representative of three independent experiments. The statistical significances were determined with Student’s *t*-test. The significance level was defined as *p* < 0.05 (^∗^).

### Western Blot Analysis

At 6, 12, and 24 h p.i., DMSO- or MG132-treated infected cells were harvested, and the pellets were resuspended in 1× lysis buffer (Cell Signaling Technology). SDS–PAGE and western blotting were performed as described previously (Huang et al., 2013b). Equal amounts of protein were subjected to SDS–PAGE and then transferred to polyvinylidene difluoride membranes. After blocking with 5% non-fat dry milk, the membranes were incubated with the primary antibodies for 2 h at room temperature, including anti-VP86 (1:1000), anti-VP136 (1:1000), anti-VP72 (1:1500), and anti-VP19 serum (1:1500). After washing with Tris buffer, the membranes were incubated for 1 h with the HRP-Goat anti-Mouse IgG (1:1000). Simultaneously, internal controls were performed by detecting β-actin protein. Immunoreactive bands were visualized with diaminobenzidine.

### 2-DE Analysis and Protein Identification

To analyze the differentially expressed proteins regulated by MG132 treatment during SGIV infection, 2-DE was performed as described previously (Zhao et al., 2010). GS cells were pretreated with either DMSO or 10 μM MG132 for 2 h, and then infected with SGIV at MOI 2. Cells were harvested and lysed in lysis buffer [20 mM Tris, 7 M urea, 2 M thiourea, 4% (w/v) CHAPS, 2 mM TBP, 0.2% IEF buffer], then protein concentrations were determined using a modified Bradford assay. All samples were stored at -80°C prior to electrophoresis. 2-DE was carried out using Immobiline strips (pI range, 3–10; GE Healthcare, Piscataway, NJ, United States), with proteins being separated according to charge, and subsequently molecular weight. The gels were stained with Coomassie brilliant blue G-250. Differentially expressed protein spots were excised from the gels for MS analysis. MALDI-TOF spectra were calibrated using trypsin autolysis peptide signals and matrix ion signals as described by Liu et al. (2013). Protein identification using peptide mass fingerprinting was performed using the MASCOT search tool (^1^Matrix Science Ltd., London, United Kingdom). Protein identification with a score >85 was regarded as positive identification.

## Results

### UPS-Related Genes Were Differently Expressed During SGIV Infection

**FIGURE 1:**
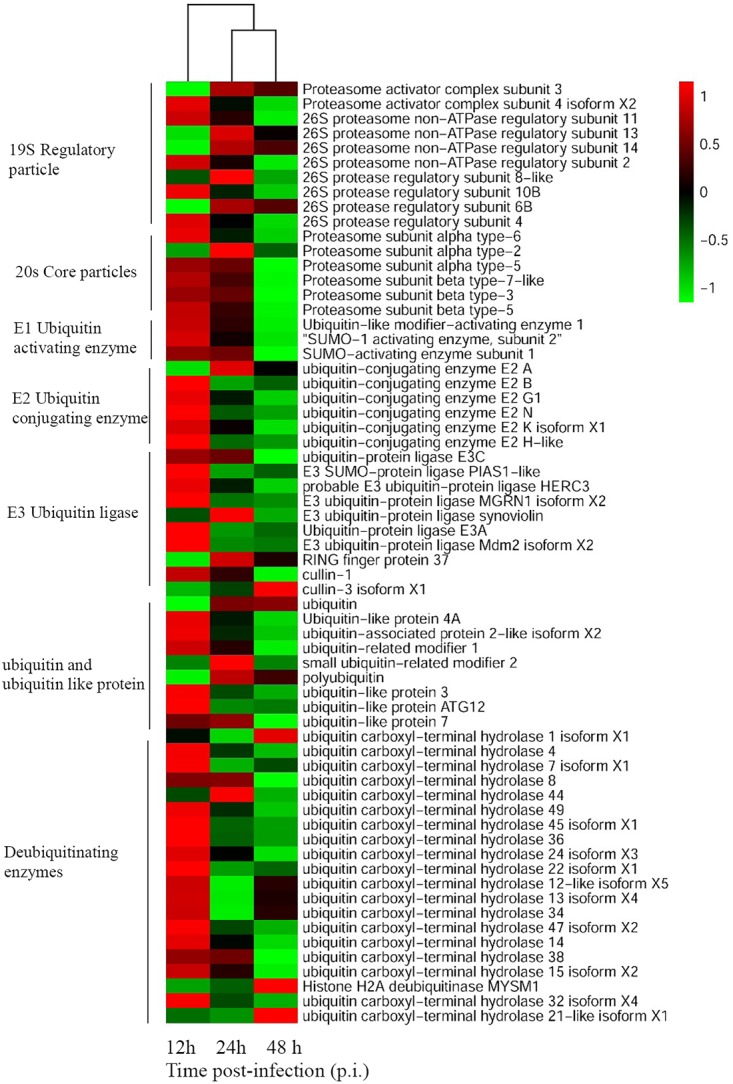
Heatmap representing gene expression of DEGs enriched to UPS during SGIV infection. Red color represents increased expression while green color represents decreased expression. Each column (12, 24, and 48 h) represents the expression levels in SGIV infected GS cells (12, 24, and 48 h) compared to mock-infected GS cells at the corresponding time points.

To unravel crucial cellular factors involved in SGIV replication, the differentially expressed cellular genes during virus infection were identified with RNA-Seq analysis. Sixty-four grouper genes related to the UPS components were differently regulated at the different stages of SGIV infection. These genes participated in various aspects of the UPS, including ubiquitination, deubiquitination and proteasome degradation. For example, the expression levels of proteasome subunit α (PSMA)2, PSMA3, ubiquitin-conjugating enzyme (UBE)2A, RING finger protein (RFP)37, ubiquitin, small ubiquitin-related modifier (SUMO)2, and ubiquitin carboxyl-terminal hydrolase (USP)1 were significantly up-regulated during SGIV infection. In contrast, the majority of genes were significantly down-regulated during SGIV infection, including ubiquitin-like modifier-activating enzyme (UBA)1, UBE2N, SUMO1, ubiquitin-like protein (UBL)3, USP36, USP4, and others listed in Figure 1.

### Multiple Proteasome Inhibitors Decreased SGIV Infection *in vitro*

**FIGURE 2:**
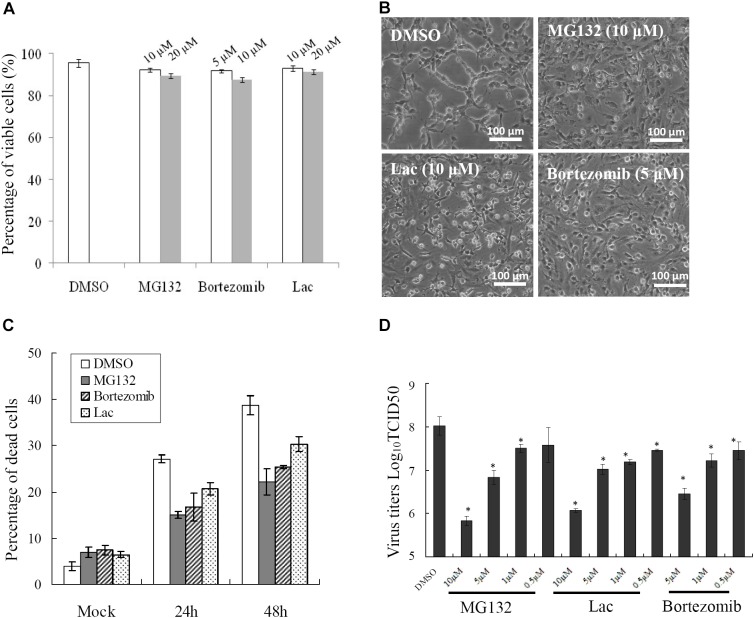
Different proteasome inhibitors reduced viral replication. **(A)** The toxicity of different proteasome inhibitors in GS cells. Effects of proteasome inhibitors on SGIV-induced CPE at 24 h p.i. **(B)**, cell death **(C)**, and viral production **(D)**. ^∗^*p* < 0.05.

To determine whether UPS was essential for SGIV infection, three structurally unrelated proteasome inhibitors, MG-132, lactacystin and bortezomib, were used to inhibit proteasome activity during SGIV infection. We evaluated the cytotoxic effects of these inhibitors on GS cells, and selected the optimal concentration of MG132 (10 μM), lactacystin (10 μM), and bortezomib (5 μM) in the following study (Figure 2A). After treatment with proteasome inhibitors, a significant delay in the severity of CPE was observed in infected cells treated with MG132, lactacystin or bortezomib, compared with that in DMSO-treated cells (Figure 2B). Given that severity of CPE evoked by SGIV is associated with cell viability (Huang et al., 2011a), we assessed virus-induced cell death under treatment with proteasome inhibitors. Consistently, treatment with these three inhibitors significantly decreased SGIV-induced cell death (Figure 2C). The effect of proteasome inhibitors on virus production was also evaluated by viral titer assay. Virus production was significantly reduced at 24 hpi in the presence of 5 or 10 μM MG132, 5 or 10 μM lactacystin or 5 μM bortezomib during infection, suggesting that the effect of proteasome inhibitors on virus production was dose dependent (Figure 2D).

### Ubiquitin-Activating Enzyme E1 and Ubiquitin Were Involved in SGIV Infection

**FIGURE 3:**
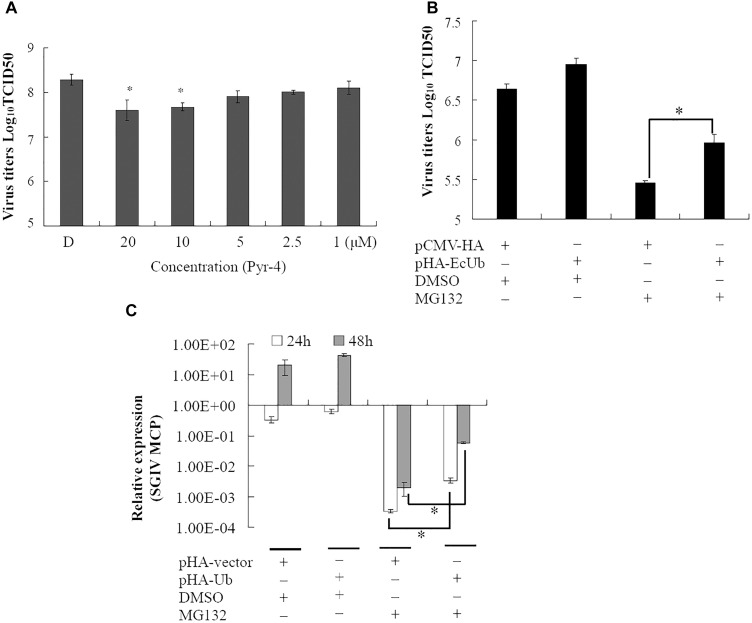
Ubiquitin-activating enzyme (E1) and ubiquitin were involved in virus infection. **(A)** The inhibitory effect of Pyr-41 on viral production was dose dependent. Over-expression of grouper ubiquitin partially countered the effect of MG132 on SGIV production **(B)** and viral gene transcription **(C)**. ^∗^*p* < 0.05.

Ubiquitin-activating enzyme E1 is one of the important components of the UPS, thus, the effect of E1 inhibitor PYR-41 on SGIV infection was also evaluated by viral titer assay. Virus production was significantly decreased in the presence of 10 or 20 μM PYR-41 (non-toxic to GS cells, data not shown) during infection (Figure 3). To determine whether inhibition of SGIV replication by MG132 was partially due to depletion of free ubiquitin, grouper ubiquitin was cloned into pCMV-HA vector as described previously (Karpe and Meng, 2012). qRT-PCR analysis indicated that the expression of ubiquitin increased significantly in recombinant plasmid pHA-EcUb overexpressing cells compared to control vector (pCMV-HA) transfected cells (data not shown). Furthermore, over-expression of pHA-EcUb partially countered the inhibitory effects of MG132, including viral production and gene transcription (Figures 3B,C). Thus, we propose that ubiquitination was also necessary for the productive infection of SGIV.

### Proteasome Inhibitor Inhibited Viral Gene Transcription and Protein Synthesis

**FIGURE 4:**
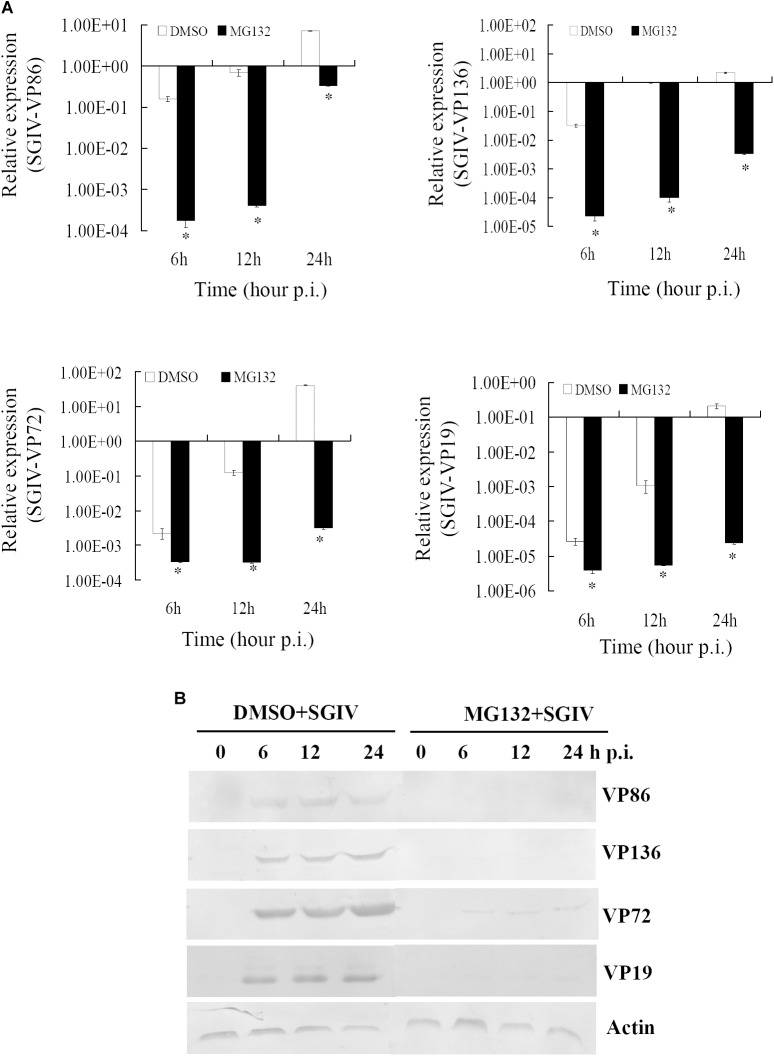
MG132 treatment significantly reduced viral gene transcription and protein synthesis. **(A)** Transcription of viral genes (immediate early gene VP86, early gene VP136, and late genes VP72 and VP19) was decreased by MG132. ^∗^*p* < 0.05. **(B)** Synthesis of viral proteins was also impaired by MG132.

To clarify the dynamic alterations of viral replication after proteasome inhibition, viral gene transcription and protein synthesis, including immediately early (VP86), early (VP136) and two late structural (VP72 and VP19) genes were examined in DMSO- or MG132-treated infected cells. At the transcription level, qRT-PCR indicated that the mRNA transcripts of VP86, VP136, VP72, and VP19 were all reduced significantly at different time points in MG132-treated infected cells comparing with the DMSO-treated cells (Figure 4A). Consistently, at the protein synthesis level, the protein products of VP72, VP19, VP136, and VP86 were obviously detected from 6 to 24 hpi during infection in DMSO-treated cells. In contrast, VP72 and VP019 were weakly detected at 6 and 24 hpi, while VP86 and VP136 were even undetectable in MG132-treated cells (Figure 4B). Our results indicated that viral gene transcription and protein synthesis during SGIV infection were severely inhibited by MG132 treatment.

### Proteasome Inhibitors Prevented Formation of Viral Factories and Transportation of Viral Proteins

**FIGURE 5:**
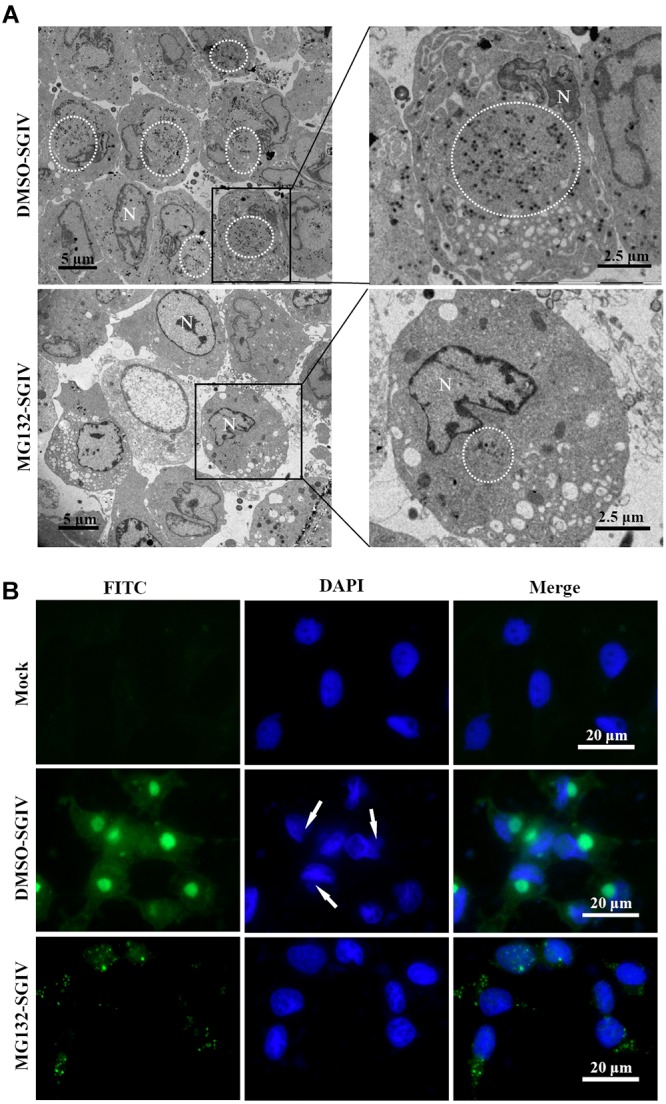
Transport of viral proteins and formation of viral factories were impaired after treatment with MG132. **(A)** Formation of viral factories after MG132 treatment. Circular places show the viral factories. N indicated nucleus. **(B)** Intracellular localization of VP019 after SGIV infection in the absence or presence of MG132. Arrows showed the virus factories.

As a large enveloped DNA virus, SGIV replicates and assembles in viral factories that form at pericentriolar sites. Under fluorescence microscopy, viral factories were observed after staining with DAPI during SGIV infection. Many viral assembly sites were observed in DMSO-treated SGIV-infected cells, but few in MG132-treated cells (Supplementary Figure S1A). To examine the ultrastructural morphology of viral factories, SGIV-infected DMSO- or MG132-treated cells were prepared for electron microscopy. Numerous viral particles were observed in almost all the cells, and the viral factories were present close to the nucleus in the majority of SGIV-infected DMSO-treated cells at 24 hpi (Figure 5A). In contrast, in MG132-treated infected cells, only a few viral particles and no factories were observed.

Cytoplasmic DNA viruses usually concentrate the structural proteins into viral assembly sites at the late stage of infection (Heath et al., 2001; Zhao et al., 2008; Huang et al., 2013a). In this study, SGIV VP19 proteins were found to be mostly localized in the viral factories in DMSO-treated infected cells at 24 h p.i. Green fluorescence spots were randomly distributed in the cytoplasm in the MG132-treated infected cells at 24 h p.i. (Figure 5B). Consistently, VP75 proteins also overlapped with viral factories in SGIV-infected DMSO-treated cells at 24 h p.i., and displayed punctate fluorescent spots in MG132-treated infected cells (Supplementary Figure S1B). Thus, our results suggested that proteasome inhibition not only prevented transportation of viral proteins, but also affected the formation of viral factories during SGIV infection.

### Proteasome Disruption Exerted More Crucial Roles at the Early Stage of SGIV Infection

**FIGURE 6:**
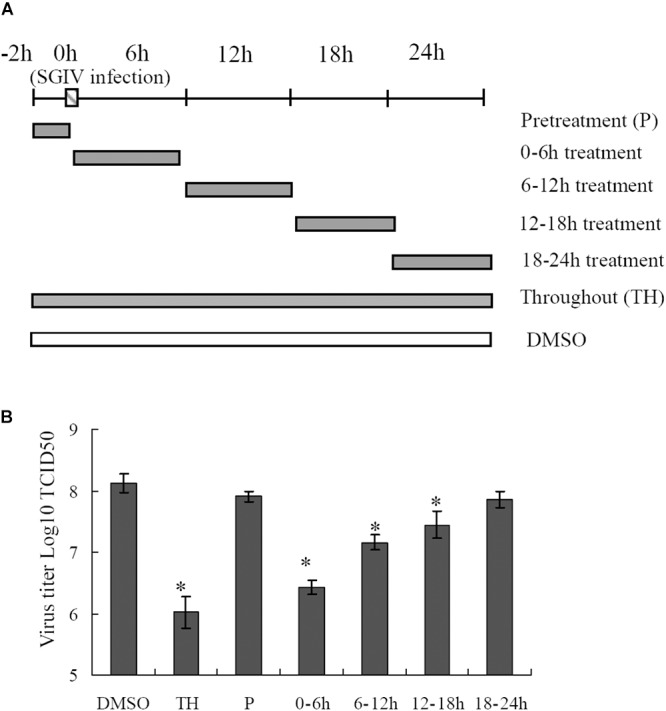
Effects of MG132 on SGIV replication at different stages of virus infection. **(A)** Experimental design for time frame experiments. GS cells were infected with SGIV and treated with MG132 at different times. The cells were washed to remove the drug and further incubated until 24 h. Whole cell lysates were collected and virus production was determined. **(B)** MG132 severely affected the virus replication at an early step during SGIV infection. ^∗^*p* < 0.05.

To delineate the potential mechanisms of proteasome on SGIV infection, reversible inhibitor MG132 was added at different times during SGIV infection as shown in Figure 6A. The virus titer assay showed that treatment with MG132 for 0–6 h resulted in significant decrease of virus production (1.6 log-unit reduction compared to DMSO treated cells). The virus titer of the group treated for 6–12 h was 1 log unit lower than that of the control. The groups treated with MG132 from 12 to 18 h p.i., and from 18–24 h p.i. showed a slight decrease of virus titer (Figure 6B), suggesting that the addition of MG132 played more important roles at the early stage of SGIV infection.

### Proteasome Inhibition Regulated Host Proteins Involved in SGIV Replication

**FIGURE 7:**
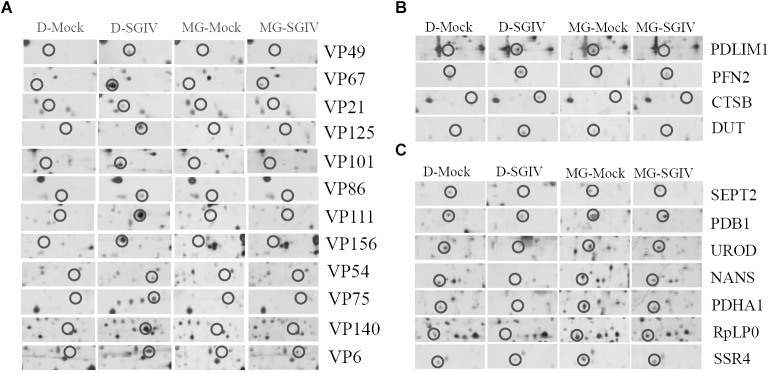
Viral or host proteins were differentially expressed in SGIV-infected cells after treatment with MG132. **(A)** Viral proteins were decreased under MG132 treatment. SGIV infection up-regulated **(B)** or down-regulated **(C)** proteins were impaired by MG132 treatment.

To investigate further the potential mechanism underlying the action of the UPS during SGIV infection, the protein samples collected from SGIV-infected and mock-infected cells in the presence or absence of MG132 were separated using 2-DE. One hundred and thirty protein spots were obviously altered in SGIV-infected cells or MG132-treated SGIV-infected cells. After MS analysis, 62 differentially expressed spots were identified, including 15 viral proteins and 47 host proteins. The identified spots were marked with numbers (Supplementary Figure S2), and the retrieved proteins corresponding to each numbered spots are listed in Supplementary Table S1. All the identified viral proteins were significantly down-regulated, and only VP67 and VP6 were weakly detectable in MG132-treated infected cells (Figure 7A). This implied that viral protein synthesis was severely decreased after proteasome inhibition.

According to protein functions and subcellular annotations from the Gene Ontology Database, the identified cellular proteins were involved in the cytoskeleton, macromolecular biosynthesis, metabolism, ubiquitin–proteasome pathway, and stress response. Among these regulated proteins, PDLIM1, PFN2, CTSB, and DUT were significantly up-regulated during SGIV infection. Interestingly, these proteins were significantly down-regulated in MG132-treated infected cells compared to mock-infected cells (Figure 7B). In contrast, SEPT2, PDB1, UROD, NAS, PDHA1, RpLP0, and SSR4 were significantly down-regulated during SGIV infection, while increased in MG132-treated infected cells compared to mock-infected cells (Figure 7C). Thus, our results suggested that certain host proteins involved in SGIV infection were regulated by proteasome inhibition.

## Discussion

The UPS can be exploited by different mammalian viruses during their life cycles, including during entry, assembly and release (Harty et al., 2001; Ott et al., 2002; Delboy et al., 2008; Kaspari et al., 2008; Tran et al., 2010; Widjaja et al., 2010; Casorla-Pérez et al., 2017). However, the potential roles of the UPS in fish viral infections remain largely uncertain (Huang et al., 2017). In our study, RNA-Seq based transcriptome analysis of SGIV-infected cells indicated that numerous genes related to the UPS were differentially regulated during SGIV infection. These genes were involved in different aspects of the UPS, including ubiquitination, deubiquitination and proteasome degradation. Proteasome subunit PSMA2, PSMA3, ubiquitin, E3 ubiquitin ligase, RFP37, UBE2A, and deubiquitinating enzyme USP1 were all significantly up-regulated during SGIV infection, suggesting that the UPS was involved in SGIV replication. During dengue virus serotype 2 infection, expression of ubiquitin-activating enzyme E1 (UBE1) and proteasome subunits were increased (Kanlaya et al., 2010). In addition, ubiquitin-conjugating enzyme, 26S proteasome regulatory subunits, and ubiquitin were also differentially regulated by tomato ringspot virus infection (Babu et al., 2008).

Although the UPS plays crucial roles during different viral infections, the underlying mechanisms are different (Delboy et al., 2008; Camborde et al., 2010; Tran et al., 2010; Greene et al., 2012). Proteasome inhibitors block avian reovirus replication at an early stage in the viral life cycle, but do not affect entry and internalization (Chen et al., 2008). The UPS is essential at all stages of human cytomegalovirus infection (Kaspari et al., 2008). In our study, both proteasome inhibitors and ubiquitin-activating enzyme E1 inhibitor delayed CPE progression in SGIV infection and reduced the viral products. The formation of viral factories was also inhibited after proteasome destruction. Vaccinia-virus-infected, MG132-treated cells also lack viral assembly sites within the cytoplasm, which is accompanied by absence of late gene expression (Teale et al., 2009). Over-expression of grouper ubiquitin partly reverses the inhibitory effect of MG132 on SGIV replication, suggesting that ubiquitin also plays crucial roles in SGIV replication, like other mammalian viruses (Si et al., 2008; Karpe and Meng, 2012; Cheng et al., 2014). As two separate arms of the UPS, ubiquitylation, and proteasomal degradation are closely linked and act at different stages (Greene et al., 2012). Therefore, we propose that the UPS is required for fish iridovirus infection *in vitro*.

As a major intracellular protein degradation system, the UPS is involved in a variety of fundamental cellular processes, including regulation of gene transcription and cell signaling, cell cycle, and cell proliferation and differentiation (Yao and Ndoja, 2012). Using 2-DE and MS analysis, we identified 62 differentially expressed proteins, including 15 viral proteins and 47 host proteins after MG132 treatment. Consistent with the data from western blotting, all the identified viral proteins were found in SGIV-infected cells, and almost undetectable in SGIV-infected MG132-treated cells, suggesting that viral protein synthesis were impaired after MG132 treatment. Apart from the viral proteins, certain host cellular proteins involved in different cell processes were regulated by SGIV infection or MG132 treatment. Among them, PDLIM1, PFN2, CTSB, and DUT were significantly up-regulated during SGIV infection, but significantly down-regulated in SGIV-infected MG132-treated cells. In contrast, SEPT2, PDB1, UROD, NAS, PDHA1, RpLP0, and SSR4 were significantly down-regulated during SGIV infection, but only slightly decreased in SGIV-infected MG132-treated cells. It has been reported that CTSB aggravates CVB3-induced viral myocarditis, probably through activating the inflammasome and promoting pyroptosis (Wang et al., 2018). Depletion of SEPT2 in HeLa cells increases replication of VACV (Beard et al., 2014). Our previous studies also demonstrated that grouper CTSB was involved in SGIV replication and SGIV induced apoptosis (Wei et al., 2014). Whether the action of CTSB on SGIV infection was mediated by UPS still remained uncertain. In addition, PDLIM1 negatively regulates nuclear factor (NF)-κB-mediated signaling, and PFN2 encodes an actin-binding protein involved in endocytosis (Ono et al., 2015; Luscieti et al., 2017). Whether these proteins exerted similar roles in SGIV infection and were regulated by UPS needs further investigation.

In summary, we reported the actions of the UPS in the life cycle of SGIV. Numerous genes related to the UPS were up/down-regulated during SGIV infection, and UPS destruction impaired SGIV replication, as demonstrated by the decrease in viral gene transcription, protein synthesis and formation assembly sites. MG132 treatment regulated certain cellular proteins that were involved in viral infection, suggesting that the UPS plays crucial roles during SGIV infection via regulation of host proteins. Thus, our study provides new insights into understanding the underlying molecular mechanism of the UPS during SGIV infection.

## Author Contributions

XH and YH carried out the main experiments, analyzed the data, and drafted the manuscript. SW and SN performed the viral titer assay and participated in the qRT-PCR experiments. YH and QQ designed the experiments and reviewed the manuscript. All authors read and approved the final manuscript.

## Conflict of Interest Statement

The authors declare that the research was conducted in the absence of any commercial or financial relationships that could be construed as a potential conflict of interest.

## Footnotes

1 http://www.matrixscience.com/

## Supplementary Material

FIGURE S1 **(A)** MG132 reduced formation of viral assembly sites. Viral assembly sites in SGIV-infected, DMSO- or MG132-treated GS cells were stained with DAPI and observed under fluorescence microscopy. **(B)** Transport of viral structural protein VP75 was impaired by MG132. Arrows indicate the viral assembly sites.

FIGURE S2 Protein expression profiles of the SGIV-infected and mock-infected cells in the presence or absence of MG132. The differentially expressed protein spots are marked with numbers for identification.

TABLE S1 Altered proteins in Mock or SGIV-infected GS cells.
