# Supplementary figures and images for: Ubiquitin–Proteasome System Is Required for Efficient Replication of Singapore Grouper Iridovirus
Source: Front Microbiol. 2018 Nov 26;9:2798. doi: 10.3389/fmicb.2018.02798 (PMC6275174; doi:10.3389/fmicb.2018.02798)

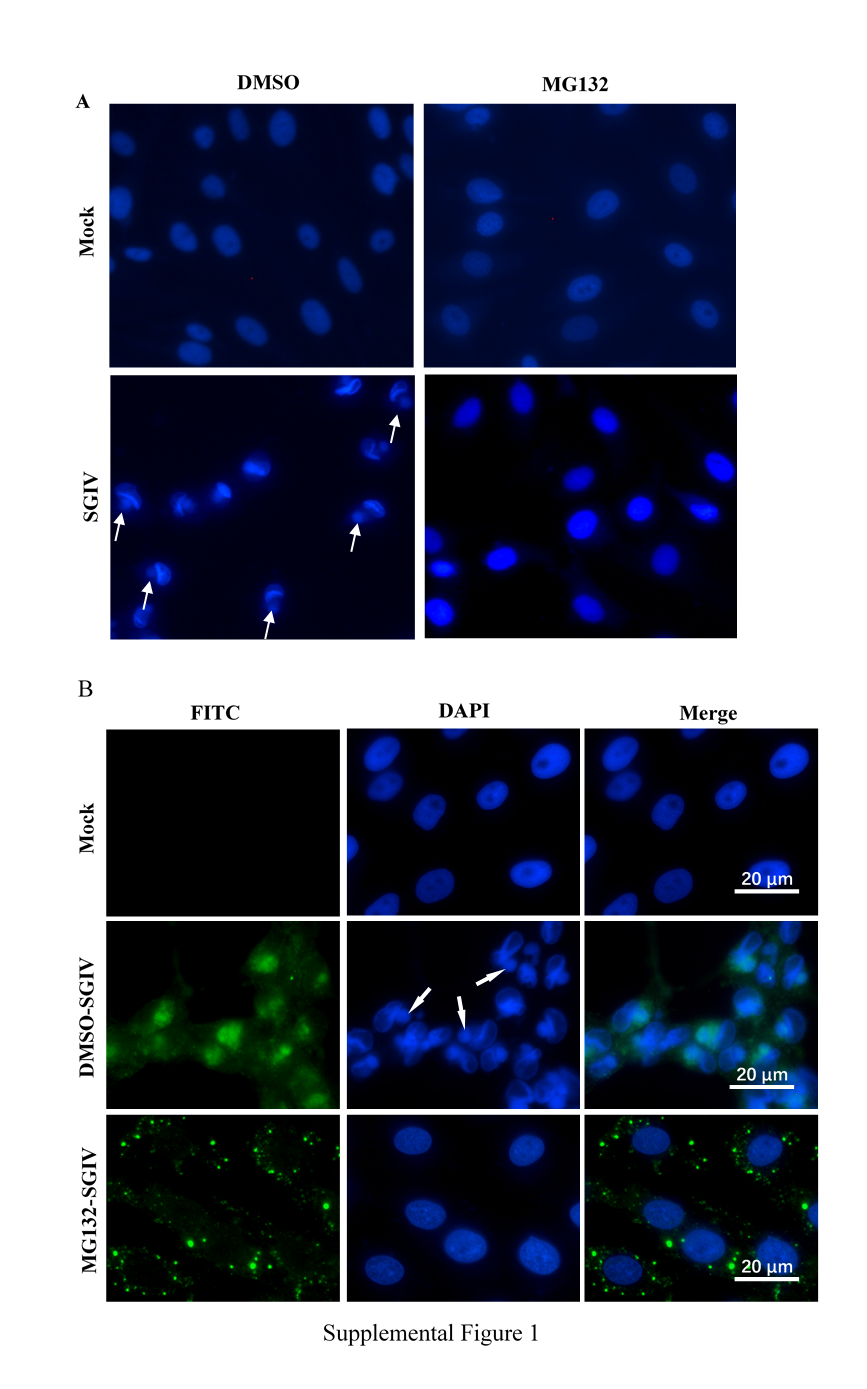

Supplement: FIGURE S1 — (A) MG132 reduced formation of viral assembly sites. Viral assembly sites in SGIV-infected, DMSO- or MG132-treated GS cells were stained with DAPI and observed under fluorescence microscopy. (B) Transport of viral structural protein VP75 was impaired by MG132. Arrows indicate the viral assembly sites. [file Image_1.TIF]

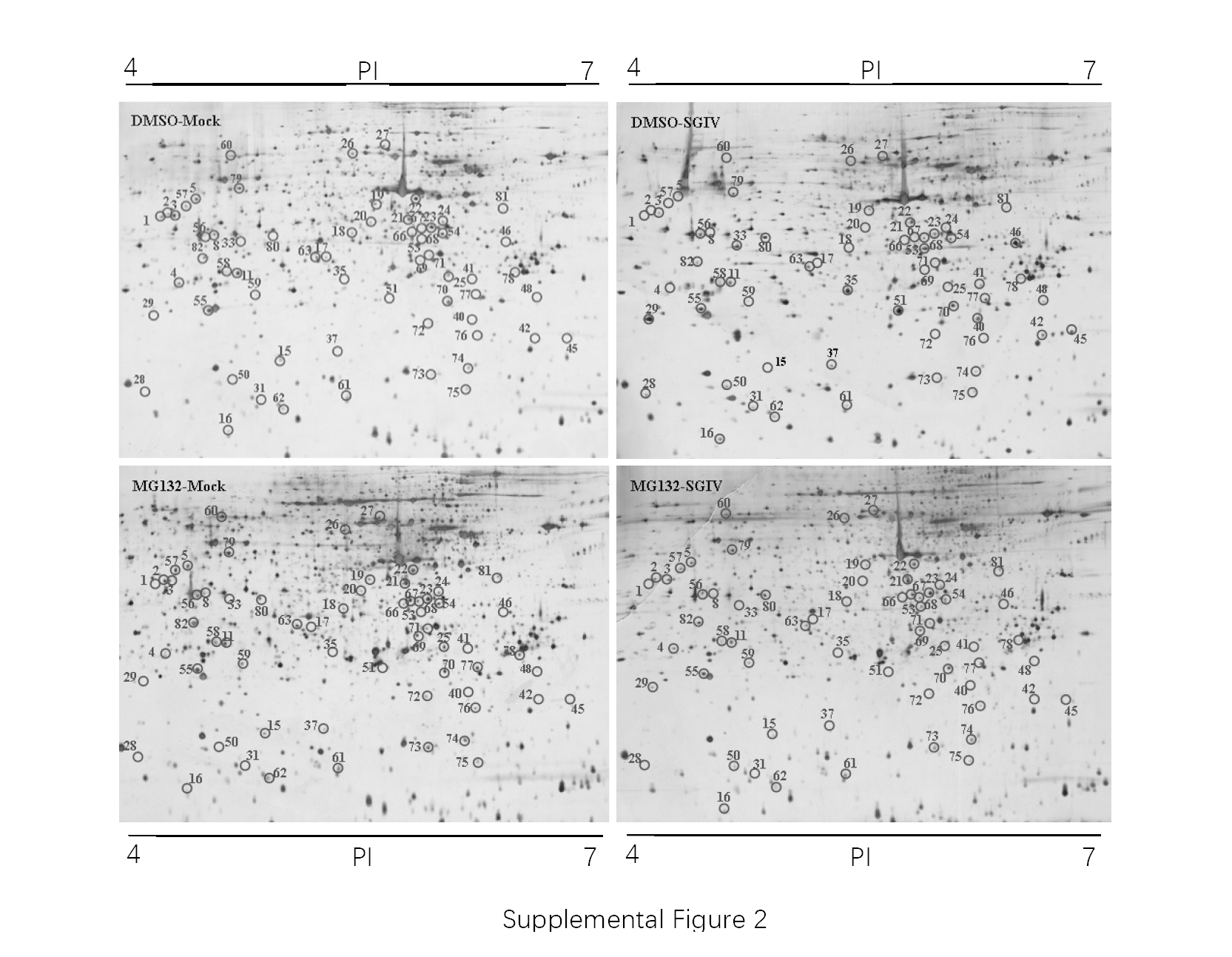

Supplement: FIGURE S2 — Protein expression profiles of the SGIV-infected and mock-infected cells in the presence or absence of MG132. The differentially expressed protein spots are marked with numbers for identification. [file Image_2.JPEG]
