# Supplementary material for: Ubiquitin–Proteasome System Is Required for Efficient Replication of Singapore Grouper Iridovirus
Source: Front Microbiol. 2018 Nov 26;9:2798. doi: 10.3389/fmicb.2018.02798 (PMC6275174; doi:10.3389/fmicb.2018.02798)
Supplement: TABLE S1 — Altered proteins in Mock or SGIV-infected GS cells. [file Table_1.DOC]

SUPPLEMENTAL TABLE 1. The altered proteins in Mock or SGIV-infected GS cells identified by PMF and MS/MS.

| Spot  No^a^. | Accession  Number^b^ | Protein name | Abbr. | MASCOT  Score^c^ | Mr (kD) /pId | Sequence  coverage (%)^d^ | Species  Name |
| --- | --- | --- | --- | --- | --- | --- | --- |
| 1 | gi|73971430 | sialic acid synthase isoform 1 | [NANS](http://www.uniprot.org/uniprot/W6FSS0) | 160 | 40.7/6.44 | 6 | Canis lupus familiaris |
| 2 | gi|21397263 | Putative phosphoserine aminotransferase | serC | 141 | 45.3/8.53 | 18 | Oryza sativa Japonica Group |
| 3 | gi|225706208 | Transaldolase | TALDO1 | 358 | 37.9/6.48 | 30 | Osmerus mordax |
| 4 | gi|323649964 | proteasome subunit beta type-7 precursor | PSMB7 | 200 | 26/8.13 | 12 | Perca flavescens |
| 5 | gi|348537496 | uroporphyrinogen decarboxylase-like | UROD | 314 | 41.6/5.98 | 19 | Oreochromis niloticus |
| 8 | gi|348513707 | crk-like protein-like | [CRKL](http://www.uniprot.org/uniprot/P46109) | 142 | 34/6.09 | 29 | Oreochromis niloticus |
| 11 | gi|47086835 | adenylate kinase 2 | AK2 | 176 | 26.9/5.53 | 31 | Danio rerio |
| 15 | gi|45709377 | Sept2 protein | SEPT2 | 396 | 44.1/5.96 | 25 | Danio rerio |
| 16 | UN_17077 | profilin-2-like | pfn2 | 126 | 33.3/8.92 | 12 | Epinephelus coioides |
| 17 | gi|229366390 | F-actin-capping protein subunit beta | CAPZB | 589 | 30.8/5.5 | 47 | Anoplopoma fimbria |
| 18 | gi|37779060 | annexin max3 | Anxa3 | 115 | 21.8/8.7 | 22 | Pagrus major |
| 19 | gi|348531962 | heterogeneous nuclear ribonucleoprotein A/B-like | HNRNPA/B | 131 | 35.5/5.52 | 9 | Oreochromis niloticus |
| 20 | gi|327281238 | OCIA domain-containing protein 1-like | [OCIAD1](http://www.uniprot.org/uniprot/Q56VL3) | 130 | 27.4/8.29 | 20 | Anolis carolinensis |
| 21 | gi|348503960 | transcriptional activator protein Pur-beta-like | PURB | 134 | 33.4/5.42 | 12 | Oreochromis niloticus |
| 22 | gi|221048057 | type I keratin | KRT1 | 311 | 30.1/6.1 | 33 | Epinephelus coioides |
| 23 | gi|2293577 | acidic ribosomal phosphoprotein PO | RPLP0 | 85 | 32.5/5.28 | 3 | Bos taurus |
| 24 | gi|348517640 | eukaryotic translation initiation factor 2 subunit 1-like | EIF2S1 | 339 | 36.3/4.98 | 51 | Oreochromis niloticus |
| 25 | gi|348544406 | grancalcin-like | [GCA](http://www.uniprot.org/uniprot/P28676) | 154 | 25.4/4.98 | 14 | Oreochromis niloticus |
| 26 | gi|348533734 | histidyl-tRNA synthetase, cytoplasmic-like isoform 1 | HARS | 240 | 58.3/5.64 | 25 | Oreochromis niloticus |
| 27 | gi|348501144 | ubiquitin carboxyl-terminal hydrolase 14-like | USP14 | 121 | 56.1/5.23 | 19 | Oreochromis niloticus |
| 28 | gi|56692686 | ORF049R | dUTPase | 464 | 17.1/6.1 | 64 | Singapore Grouper Iridovirus (SGIV) |
| 29 | gi|56692704 | ORF067L | VP67 | 639 | 21.7/6.43 | 84 | SGIV |
| 31 | gi|56692658 | ORF021L | VP21 | 275 | 16.5/6.15 | 69 | SGIV |
| 33 | gi|56692738 | ORF101R | VP101 | 500 | 35.2/6.11 | 46 | SGIV |
| 35 | gi|56692793 | ORF156L | VP156 | 431 | 31.2/5.6 | 58 | SGIV |
| 37 | gi|229368092 | Deoxyuridine 5-triphosphate nucleotidohydrolase | [DUT](http://www.uniprot.org/uniprot/P33316) | 222 | 18.9/6.82 | 21 | Anoplopoma fimbria |
| 40 | gi|56692762 | ORF125R | VP125 | 309 | 21.6/5.91 | 68 | SGIV |
| 41 | gi|56692691 | ORF054R | VP54 | 199 | 25.2/5 | 28 | SGIV |
| 45 | gi|56692712 | ORF075R | VP75 | 591 | 19.9/4.5 | 48 | SGIV |
| 46 | gi|56692777 | ORF140R | VP140 | 365 | 32.2/4.85 | 60 | SGIV |
| 48 | gi|327239610 | cathepsin B | CTSB | 123 | 18.9/4.66 | 14 | Epinephelus coioides |
| 50 | gi|56692723 | putative immediate-early protein | VP86 | 198 | 17.2/7.88 | 32 | SGIV |
| 51 | gi|56692748 | ORF111R | VP111 | 478 | 29.7/5.47 | 33 | SGIV |
| 53 | gi|56692643 | ORF006R | VP6 | 154 | 29.5/5.27 | 11 | SGIV |
| 54 | gi|348532149 | ubiquitin carboxyl-terminal hydrolase isozyme L5-like isoform 1 | UCH-L1 | 312 | 37.9/5.1 | 37 | Oreochromis niloticus |
| 55 | gi|60688276 | Prdx3 protein | PRDX3 | 348 | 27.2/8.89 | 27 | Danio rerio |
| 56 | gi|213511436 | PDZ and LIM domain protein 1 | PDLIM1 | 318 | 34.4/5.83 | 36 | Salmo salar |
| 57 | gi|548408 | Pyruvate dehydrogenase E1 component subunit alpha | PDHA1 | 124 | 39/5.54 | 12 | [Acholeplasma laidlawii](http://www.ncbi.nlm.nih.gov/Taxonomy/Browser/wwwtax.cgi?lvl=0&id=2148) |
| 58 | gi|41393129 | chloride intracellular channel protein 4 | [CLIC4](http://www.google.com.hk/url?sa=t&rct=j&q=&esrc=s&source=web&cd=1&cad=rja&uact=8&ved=0CCAQFjAA&url=http%3A%2F%2Fwww.uniprot.org%2Funiprot%2FQ9Y696&ei=afCHVeT8HYq2uATl3oO4Aw&usg=AFQjCNHmGrl9C2adM0Diyf36IKU92kKYZw&bvm=bv.96339352,d.c2E) | 159 | 28.6/5.71 | 31 | Danio rerio |
| 59 | gi|348511213 | growth factor receptor-bound protein 2-like | GRB2 | 150 | 25.4/5.59 | 14 | Oreochromis niloticus |
| 60 | gi|348524895 | T-complex protein 1 subunit alpha-like | TCP1 | 307 | 60.9/6.19 | 17 | Oreochromis niloticus |
| 61 | gi|380449367 | thioredoxin domain-containing protein 12 | TXNDC12 | 110 | 19.4/5.12 | 27 | Epinephelus coioides |
| 62 | gi|119616603 | mago-nashi homolog, isoform CRA_b | MAGOH | 204 | 10.8/6.28 | 46 | Homo sapiens |
| 63 | gi|348520068 | purine nucleoside phosphorylase-like | PNPase | 261 | 32.6/6.12 | 37 | Oreochromis niloticus |
| 66 | gi|163516 | protein phosphatase type 2A catalytic subunit | [PPP2CB](http://en.wikipedia.org/wiki/PPP2CB) | 98 | 37.9/5.46 | 4 | Bos taurus |
| 67 | gi|164691025 | ribosomal protein LP0 | RpLP0 | 275 | 34.3/5.2 | 23 | Solea senegalensis |
| 68 | gi|348532057 | 2-oxoisovalerate dehydrogenase subunit beta | [BCKDHB](http://www.uniprot.org/uniprot/P21953) | 89 | 42.9/6.26 | 5 | Oreochromis niloticus |
| 69 | gi|225708302 | Proteasome subunit alpha type 3 | PSMA3 | 202 | 28.7/4.96 | 27 | Osmerus mordax |
| 70 | UN_grouper_19824 | calpain | CAPN1 | 395 | 26.9/5.72 | 40 | Epinephelus coioides |
| 71 | gi|348526264 | COP9 signalosome complex subunit 7a-like | SGN7a | 274 | 29.7/5.17 | 27 | Oreochromis niloticus |
| 72 | gi|229366520 | RNA-binding protein 8A | [RBM8A](http://www.uniprot.org/uniprot/Q9Y5S9) | 153 | 19.8/5.65 | 27 | Anoplopoma fimbria |
| 73 | gi|225708130 | Ferritin, heavy subunit | [FTH1](http://www.uniprot.org/uniprot/P02794) | 99 | 20.7/5.23 | 18 | Osmerus mordax |
| 74 | gi|334362338 | translocon-associated protein subunit delta precursor | [SSR4](http://www.uniprot.org/uniprot/P51571) | 387 | 18.2/5.27 | 30 | Epinephelus coioides |
| 75 | gi|229366762 | Baculoviral IAP repeat-containing protein 5 | BIRC5 | 104 | 16.9/4.94 | 23 | Anoplopoma fimbria |
| 76 | gi|348522797 | chromobox protein homolog 3-like | [CBX3](http://www.uniprot.org/uniprot/Q13185)-like | 189 | 20.2/4.86 | 27 | Oreochromis niloticus |
| 77 | gi|291190540 | calpain small subunit 1 | capns1b | 139 | 24.7/5.14 | 19 | Salmo salar |
| 78 | gi|229367530 | Proteasome subunit alpha type-5 | [PSMA5](http://www.uniprot.org/uniprot/P28066) | 209 | 26.5/4.74 | 30 | Anoplopoma fimbria |
| 79 | gi|348537840 | cytochrome b-c1 complex subunit 2 | [UQCRC2](http://www.uniprot.org/uniprot/P22695) | 184 | 48.7/7.09 | 9 | Oreochromis niloticus |
| 80 | gi|348524661 | DNA-(apurinic or apyrimidinic site) lyase-like | APEX1 | 153 | 35.1/6.28 | 19 | Oreochromis niloticus |
| 81 | gi|348533059 | hsp70-binding protein 1-like | HSPBP1 | 177 | 38.3/4.78 | 12 | [Oreochromis niloticus](http://www.ncbi.nlm.nih.gov/Taxonomy/Browser/wwwtax.cgi?lvl=0&id=8128) |
| 82 | [gi|162448150](http://www.matrixscience.com/cgi/protein_view.pl?file=..%2Fdata%2F20120410%2FFtGAobenT.dat&hit=1&db_idx=1) | pyruvate dehydrogenase E1 component subunit beta | PDB1 | 383 | 35.7/6.04 | 48 | Acholeplasma laidlawii |

*a* the spot numbers (Spot No.) corresponded to the same numbers as indicated in Supplemental Figure 2.

*b* Accession no.was the MASCOT result of MALDI-TOF/MS searched from the NCBI database.

*c* PMF Protein Mass Fingerprint Score/matched peptides. (PMF score > 85 significant).

*d* Sequence coverage (%) is the number of amino acids spanned by the assigned peptides divided by the sequence length.
